# Mindfulness Group Visits for Chronic Low Back Pain in Primary Care: The OPTIMUM Pragmatic Clinical Trial

**DOI:** 10.1093/geroni/igaf122.2126

**Published:** 2025-12-31

**Authors:** Natalia Morone, Tra Nguyen, Keturah Faurot, Susan Gaylord, Kathleen McTigue, Kristina Astone, Janice Weinberg, Carol Greco

**Affiliations:** Boston University - Boston Medical Center, Boston, Massachusetts, United States; Boston Medical Center, Boston, Massachusetts, United States; The University of North Carolina at Chapel Hill, Chapel Hill, North Carolina, United States; The University of North Carolina at Chapel Hill, Chapel Hill, North Carolina, United States; University of Pittsburgh, Pittsburgh, Pennsylvania, United States; Boston University, Boston, Massachusetts, United States; Boston University, Boston, Massachusetts, United States; University of Pittsburgh, Pittsburgh, Pennsylvania, United States

## Abstract

Mindfulness is recommended as a first-line non-pharmacologic treatment for chronic low back pain (cLBP). However, accessibility is challenging. The OPTIMUM trial (Optimizing Pain Treatment In Medical settings Using Mindfulness) compared a telehealth-delivered group mindfulness program for primary care patients with cLBP to usual care. Here we describe the methods of this investigation, and report on baseline sample characteristics, intervention acceptability, and retention. The intervention is an 8-session program based upon Mindfulness-Based Stress Reduction (MBSR) led by an experienced MBSR instructor and co-led by a primary care provider who participated in sessions and met briefly with each participant. 451 adults with cLBP were randomized to the intervention or to usual medical care, across three sites. The primary outcome was the PEG (Pain intensity, Enjoyment, General Activity). Participants also completed measures of physical and psychological function, medications, and healthcare utilization, at baseline, week-8, and 6- and 12-months. Participants’ mean age=52.1 years (SD 14.7), 315 (70%) female, 176 (39%) are Black, 202 (45%) White, 253 (56%) not employed, 140 (31%) income < $25,000, 332 (74%) have pain ≥ 5 years duration. Participants’ pain intensity/pain interference was moderate at baseline, with PEG score=6 (SD 2.4). Retention was 89% at 12-month follow-up. OPTIMUM successfully enrolled and retained primary care patients with cLBP into a telehealth group mindfulness program. The program holds promise for integration into routine primary care medicine.

